# Calculating the Home Advantage in Soccer Leagues

**DOI:** 10.2478/hukin-2014-0001

**Published:** 2014-04-09

**Authors:** Miguel-Ángel Gómez, Richard Pollard

**Affiliations:** 1 Faculty of Physical Activity and Sport Sciences, Polytechnic University of Madrid, Spain.; 2 Department of Statistics, California Polytechnic State University, San Luis Obispo, USA.

## Abstract

A recent article published in the Journal of Human Kinetics (Saavedra et al., 2013) was based on a flawed methodology when calculating the home advantage values in soccer leagues. This led to incorrect calculations, false conclusions and some misleading results about home advantage in 52 soccer leagues of UEFA countries over a 10 year period. The aim of this letter was to explain these flaws and to make sure future research would not be influenced by the subsequent results and conclusions that had been presented

## Problems with the calculation of home advantage in soccer

In the Material and Methods section, under Procedures, on page 143, the authors stated that home advantage was calculated by dividing points won at home by total points won, home and away. This would have been fine, but it was not what they proceeded to do. The home advantage values reported in Tables 1, 2, 3 and 4 were obtained by dividing points won at home by three times the total number of games played. The authors appeared to forget that when a game was drawn a total of only two points were awarded, as opposed to three when a game ended in a win for one of the teams. This led to some misleading results. For example the authors claimed that there was a home *disadvantage* in Lithuania (49.09%), Latvia (49.85%), Estonia (49.41%), Wales (49.99%), Malta (48.03%) and Northern Ireland (48.80%) when in fact more games were won at home than away in each of these countries (see Table 2, page 145). The authors stated that they had used the procedure developed by Pollard (1986) which was based on the now universally mandated system of awarding each team three points for a win, zero points for a loss, and one point each team for a draw. The procedure had been used in many previous studies on home advantage in football (Pollard, 2006a, 2006b; Pollard and Gómez, 2009, 2012; Pollard et al., 2008; Sánchez et al., 2009; Seckin and Pollard, 2008), most of which were, in fact, cited by Saavedra et al. (2013). Had the correct procedure been employed, the home advantage values for the countries mentioned above would have been as follows: Lithuania (53.20%), Latvia (53.05%), Estonia (52.16%), Wales (53.87%), Malta (51.42%) and Northern Ireland (52.82%), each indicating a small home *advantage*, and certainly not a disadvantage. Since all home advantage values in Tables 1, 2, 3 and 4 are incorrect, the rest of the paper is invalid and the numerical results can be disregarded.

## The relationship between team quality and home advantage

The study reported by Saavedra et al. (2013) also included a claim that better quality teams had higher home advantage, a dubious conclusion as no analysis on individual teams, as opposed to leagues, appears to have been done. It is also a conclusion in direct contradiction to that found in other studies (Pollard and Gómez, 2009, 2012). In fact, the high positive correlation between home advantage and team quality (see page 147) is hard to believe, since strong teams win most of their games (both at home and away) and thus cannot achieve high home advantage values when the quantification of home advantage is based on a comparison of points won at home and away. It is impossible to assess what the authors did there, since they gave no indication of how the reported correlation of 0.721 was obtained, other than to say that it was the correlation between points gained by a team with home advantage. Was this based on the home advantage of every team in every league over each of the 10 seasons analyzed, well over 5,000 pairs of values? If so, what adjustments were made for points won when countries had different numbers of teams and played different numbers of games?

Finally, we reiterate that the purpose of this letter was to alert readers to the fact that the results and conclusions of the paper by Saavedra et al. (2013) could not be considered reliable since they were based on faulty analysis.
